# Supportive supervision for medicines management in government health facilities in Kiambu County, Kenya: a health workers’ perspective

**DOI:** 10.11604/pamj.2015.20.237.5872

**Published:** 2015-03-13

**Authors:** Oscar Otieno Agoro, Ben Onyango Osuga, Maureen Adoyo

**Affiliations:** 1 Ministry of Health, Nairobi, Kenya; 2 Kenya Methodist University, Nairobi, Kenya

**Keywords:** Supportive Supervision, Medicines, Medicines Management, Government Health Facilities

## Abstract

**Introduction:**

Effective supportive supervision is widely recognized as essential for optimal management of medicines in government health facilities and also in contributing towards improved access and utilization of health services. This study sought to examine the extent supportive supervision for medicines management in government health facilities from a health worker perspective.

**Methods:**

A cross-sectional study was done targeting health workers managing medicines in government health facilities in Kiambu County. One hundred and thirty eight respondents took part in the study which explored the quality of supportive supervision from a health worker's perspective, and also examined the factors influencing their contentment with the level of supervision received. A statistical analysis was done using SPSS 21 and Excel 2013.

**Results:**

Supervisory visits from all levels of health management were not regularly done, standard checklists were not routinely used, and action plans irregularly developed and followed up. Only 54 (38.6%) respondents were satisfied with the levels of supportive supervision that they received, with satisfaction significantly differing across the professional cadres, *χ*^*2*^ (12, n = 138) = 29.762, *p* = .003; across the different tiers of health facilities, *r*_*s*_ (138) = 0.341, *p* < .001; and with the education levels of the respondents, *r*_*s*_ (138) = 0.381, *p* < .001.

**Conclusion:**

The study concluded that supportive supervision for medicines management that government health facilities received was still inadequate, and health workers were dissatisfied with the level of supervision that they received. The study recommends a review of the support supervision policy at the county level to address the unearthed inefficiencies and improve supervision for medicines management in government health facilities.

## Introduction

Supportive supervision is widely recognized as essential for improving health worker performance and achieving the health Millennium Development Goals. It is a process whereby managers and supervisors guide and encourage personnel to optimize their performance in a supportive environment and recognize them when they attain a high level of performance [1]. In supportive supervision the supervisor works closely with people he or she supervises to establish goals, monitor progress and identify opportunities for improvement. If carried out properly, supportive supervision has been shown to lead to higher health worker motivation, increased and sustained job satisfaction, improved service quality as staff learn and improve skills on-the-job, efficient use of resources as staff are supported to prioritize activities and allocate resources accordingly and enhanced equity in access to services, as staff are reminded of the health needs of the population and encouraged to work towards meeting these needs [2, 3]. Adequate supportive supervision requires that health facilities are regularly visited, and that both teams are aware of the scheduling of the visits. The quality of the visits should also be ensured through the development of supportive supervision policies and the consistent use of the standard tools developed for the exercise such as checklists. Action plans should also be jointly crafted at the end of each supervisory visit, and should be followed up in the subsequent supervisory visits to ensure continuity and implementation of the recommendations [4].

Several constraints to conducting regular supportive supervision in low and middle income countries have been identified by several studies. These challenges include restricted mobility of supervisors that constrains field supervision, lack of “supportive” skills due to lack of training, absence or lack of use of standard checklists during supervision, failure to develop and follow up on action plans, vertical programs with vertical supervision lead to fragmentation, lack of clear guidelines for supportive supervision, and absence of a supportive supervision policy in health systems [5–8]. Studies have called on the need for further additional research to establish how supportive supervision in health systems should be best carried out effectively since good performance by the peripheral health worker reflects on the work and integrity of the supervisor [4, 9, 10]. This study was therefore carried out to establish the extent of supportive supervision for medicines management in government health facilities in Kiambu County, Kenya. It sought to establish whether the health workers understood the existing medicine management supervision schedules, who were supposed to supervise them, and how the actual supervision was conducted. Since it was done from a health worker perspective, the influence of their different socio-demographic characteristics on the rating of satisfaction with the supervision they received was also examined. The outcome was intended to add to the existing knowledge on supportive supervision and contribute towards improving the efficiency of medicines management and service delivery in health systems of developing countries.

## Methods

**Study site:** The study was conducted in Kiambu County, Central Kenya region. The county covers approximately 2,543 Km^2^ and has 12 sub-counties namely: Gatundu North, Gatundu South, Juja, Thika Town, Ruiru, Githunguri, Kiambu Town, Kiambaa, Kabete, Kikuyu, Limuru and Lari. It has a total of 85 active government health facilities that serve a catchment population of approximately 1,732,282 people - 49% males and 51% females.

**Study design:** Cross-sectional design was used on this study that was carried out in the month of July 2014.

**Study population:** All health workers directly involved in the management of medicines in government health facilities across all tiers of care in Kiambu County formed the population. They were drawn from various cadres ranging from Pharmacists, Pharmaceutical Technologists, Clinical Officers, and Nurses.

**Sampling technique:** Cochran's formula for sample size determination from a finite population was used to establish the sample size. Proportionate stratified random sampling was then used to find the exact numbers of respondents to be sampled across the different tiers of care which formed the strata. A total of 153 respondents were targeted after a pre-adjustment of the sample size to account for an anticipated non-response [11].

**Data collection instruments:** A semi-structured questionnaire was used to collect data from the respondents. The questionnaire consisted of both open ended and closed questions, and was administered to the staff directly involved with managing medicines in the health facilities. Validity and reliability of the questionnaire were ascertained in a pilot test that was conducted among health workers in a separate county in Kenya that had characteristics similar to those of the study area. A Cronbach's Alpha Coefficient test on SPSS gave a coefficient of > 0.700.

**Data analysis:** After collecting the data, editing and sorting of the questionnaires were done to determine the level of completeness. The responses in the completed questionnaire were coded and entered into a data entry template. Data entry and analysis were performed by using SPSS for Windows version 21 and Excel 2013. Descriptive data was presented in summary tables and graphs. Analyses to test hypotheses were performed using Pearson's Chi-Square analyses for nominal scale data. Spearman's Rank Order correlation and Pearson's correlation were done for variables measured on ordinal and interval scales, and the correlation coefficients calculated to determine the intensity and direction of the relationships between the variables. The level of signification was set at a *p* value less than 0.05.

**Legal and ethical considerations:** The research clearance was obtained from the Kenya Methodist University Research and Ethics department covering the area of study. The protocol required in order to collect data from the health facilities was also observed. The County Chief Officer of Health and all Sub County Medical Officers of Health were informed, and their consent to conduct the study was obtained. Consent was also obtained from the study respondents as participation was strictly voluntary. Benefits of the study were explained to the participants before they responded to the questionnaires. All the respondents were assured of confidentiality of all the information they and their names were not be recorded so as to protect their identity.

## Results

Of the 153 health workers targeted in the study, 138 responded to the questionnaires giving a response rate of 90.2%. There were slightly more females 72 (52.2%) than males 66 (47.8%) in the sampled respondents who were composed of Pharmacists, Pharmaceutical Technologists, Nurses and Clinical Officers. The respondents were drawn from facilities at the different tiers of care due to the stratified nature of the sampling employed. Most of them were diploma holders 89 (64.5%) as shown in Table 1. Whereas most respondents expected a supervisory visit from the National Health Management Team (NHMT) semi-annually 73 (52.9%) and annually 47 (34.1%), the actual visits by the NHMT were mostly irregular 88 (63.8%) with 42 (30.4%) of the visits reported to be annual according to the respondents. Most respondents expected the County Health Management Teams (CHMT) to conduct its supervisory visits quarterly 43 (31.2%) and semi-annually 41 (29.7%). Actual visits by the CHMT were reported to be irregular by a majority of the respondents 104 (75%) in the period prior to the study. A majority of the respondents 110 (79.7%) reported that they expected the Sub-County Health Management Teams (SCHMT) to conduct medicine management supervision in their respective health facilities quarterly. The actual visits were however split between quarterly 73 (52.9%) and irregularly 56 (40.6%) according to the respondents. Facility Health Management Teams (FHMT) on the other hand were expected by most respondents 92 (66.7%) to be conducting monthly medicine management supervisory visits. The actual visits were however split between monthly 69 (50%) and irregular visits 56 (40.6%) according to the respondents Table 2.

**Table 1:** Socio-demographic characteristics of respondents (n = 138)

Variable	Frequency (%)
Gender	
Female	72 (52.2)
Male	66 (47.8)
Cadre	
Pharmacist	41 (29.7)
Pharmaceutical Technologist	34 (24.6)
Nurse	48 (34.8)
Clinical Officer	15 (10.9)
Education Level (Highest)	
Masters	5 (3.6)
Bachelors	38 (27.5)
Diploma	89 (64.5)
Certificate	6 (4.3)
Respondents’ health facility level	
Level 5	13 (9.4)
Level 4	35 (25.4)
Level 3	36 (26.1)
Level 2	54 (39.1)

**Table 2:** Supportive supervision: expected versus actual (n = 138)

Supervisory Team	Monthly	Quarterly	Semi-Annually	Annually	Irregularly
**National HMT**					
Expected	0 (0)	13 (9.4)	73 (52.9)	47 (34.1)	5 (3.6)
Actual	0 (0)	2 (1.4)	6 (4.3)	42 (30.4)	88 (63.8)
**County HMT**					
Expected	2 (1.4)	43 (31.2)	41 (29.7)	2 (1.4)	50 (36.2)
Actual	0 (0)	18 (13)	16 (11.6)	0 (0)	104 (75)
**Sub County HMT**					
Expected	15 (10.9)	110 (79.7)	3 (2.2)	0 (0)	10 (7.2)
Actual	6 (4.3)	73 (52.9)	2 (1.4)	1 (0.7)	56 (40.6)
**Facility HMT**					
Expected	92 (66.7)	13 (9.4)	2 (1.4)	1 (0.7)	30 (21.7)
Actual	69 (50)	10 (7.2)	2 (1.4)	1 (0.7)	56 (40.6)

**Figure 1:**
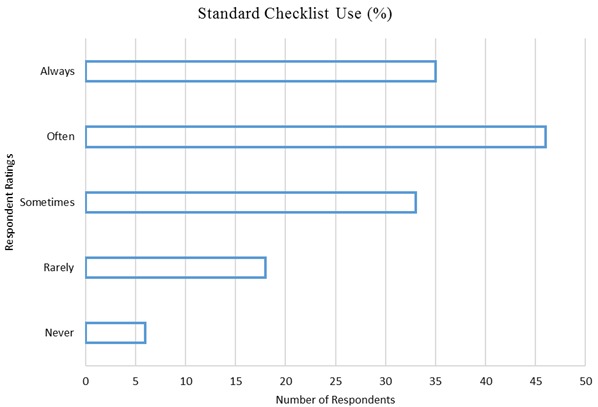
Use of standard supervision checklists (n = 138)

Most of the respondents 108 (78.3%) reported that they had had their most recent supervisory visit within the last month as of the study date while 27 (19.6%) had their most recent supervisory visit within the last three months. Three (2.2%) respondents reported that they had never been visited for medicine management supportive supervision by any team in the last one year. The mean travel distance between the respondents’ health facilities and their CHMT offices was 42.06 kilometers (SD= 19.15) while that between their health facilities and the SCHMT offices was 8.44 kilometers (SD = 9.58). The frequency of supervisory visits by the CHMT was found to be independent of the health facility travel distance from the CHMT offices, *r* (138) = 0.051, p= .555. The frequency of supervisory visits by the SCHMT was also found to be independent of the health facility travel distance from the SCHMT offices, *r* (138)= - 0.090, p= .291. The use of standard checklists by the supervisors that guide the supervision exercise was reported as often to always done by 81 (57%) respondents while 33 (23.9%) respondents reported that checklists were sometimes used. Twenty four (17.7%) respondents said that checklists were rarely to never use while conducting the supervision exercise during the visits Figure 1. The use of checklists during supportive supervision of medicines management was found to be independent of the tier of care of the health facility visited, *r*_*s*_ (138) = - 0.028, *p* = .741.

**Figure 2:**
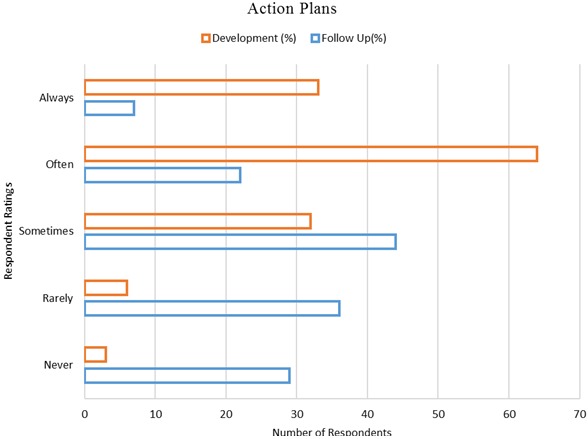
Action plan development and follow up (n = 138)

**Figure 3:**
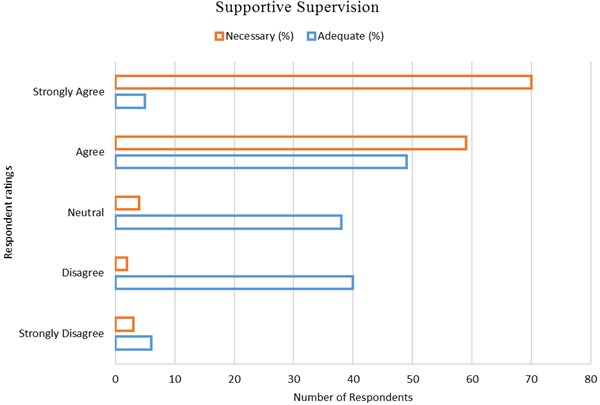
Supportive supervision – necessity and adequacy (n = 138)

Whereas written Action Plans were reported to be often to always developed by most respondents 97 (70.3%) at the end of each supervisory visit, only 29 (21%) of the respondents reported that they were often to always availed for review and follow up in the subsequent supervisory visits by the supervisors Figure 2. The development of Action Plans at the end of medicine management supervisory visits was found to be moderately correlated to the tier of care of the health facilities visited, *r*_*s*_ (138) = 0.440, *p* < .001. While a majority of the respondents 129 (93.5%) were of the opinion that regular supportive supervision was necessary for optimal management of medicines in government health facilities, only 54 (38.6%) respondents were satisfied with the level of supportive supervision for medicines management that they were receiving from their health management teams charged with that role. A portion of the respondents, 38 (27.5%) remained non-aligned when asked to rate the adequacy of the visits they received Figure 3. The rating of adequacy of supportive supervision visits by the respondents was found to be dependent on the respondents’ professional cadres, *χ* ^*2*^ (12, n = 138) = 29.762, *p* = .003; highest level of education, *r*_*s*_ (138) = 0.381, *p* < .001; and the tier of care of the health facilities where they worked, *r*_*s*_ (138) = 0.341, *p* < .001 Table 3. Contentment with the adequacy of medicine management supervisory visits by the respondents was found not to significantly vary with gender, *χ* ^*2*^ (4, n = 138) = 5.781, *p* = .216; with the duration that the respondent had served in public service, *r*_*s*_ (138) = - 0.025, *p* = .773; with the duration that the respondent had served in their current health facility, *r*_*s*_ (138) = 0.151, *p* = .077; and across the different sub-counties where the respondents’ health facility were, *χ* ^*2*^ (32, n = 138) = 0.459, *p* = .308 Table 3.

**Table 3:** Respondent characteristics and supportive supervision ratings

Respondent Characteristics	Association Coefficient Output (n = 138)
Gender	*χ* ^*2*^ *=* 5.781
Cadre	*χ* ^*2*^ *=* 29.762
Education	*r*_*s*_ *=* 0.381
Duration in Public Service	*r*_*s*_ = - 0.025
Duration in Health Facility	*r*_*s*_ = 0.151
Sub-County	*χ* ^*2*^ = 0.459
Tier of Health Facility	*r*_*s*_ = 0.341

## Discussion

The study had a high response rate of 90.2% which fell within the range of ≥ 80% for research intended to represent pharmacy-related surveys [12]. This high response rate was attributed to the manner in which the questionnaire was designed and the approach employed by the researcher. The questionnaires did not ask for information that could make the respondents feel that they could be traced from their questionnaires. The length of the questionnaire was adequate and personal information like age, duty station and telephone numbers were not asked. Consent was also sought through their respective sub-county heads and this gave them confidence on the legitimacy of the study. There were slightly more females 72 (52.2%) than males 66 (47.8%) in the sampled respondents and this appeared to contradict the findings of Newman et al., (2011) which established that the proportion of male to female medical practitioners in Kenya stood at 60% and 40% respectively. The explanation lay in the differences in the gender ratios across the different occupational cadres as was seen by Newman et al., (2011). In Kenya, 71% of Nurses are female while 65% of Pharmacists are male. Since this study had a mix of Pharmacists, Pharmaceutical Technologists, Clinical Officers and Nurses as respondents, and with Nurses accounting for about 35% of the respondents, there was the possibility of having the gender ratio slightly altered as was observed. Nurses and Clinical Officers mostly managed medicines in dispensaries and in health centres because of the shortage of pharmaceutical staff in government health facilities [5].

Despite the devolution of health services in Kenya one year before the study was conducted, health workers in the counties still expected to be supervised by the NHMT, a team whose mandate was not to conduct supportive supervision in health facilities devolved to the county governments. The study showed that a large section of health workers still did not understand the difference in the mandates of the two tiers of government as far as the health sector was concerned, a scenario that had also been observed in other studies among Indian, Nigerian and Indonesian health workers [13–15]. Respondents in the study could not clearly agree on how often they expected a supervisory visit by the CHMT and this was made worse by the irregular supervisory visits by the same team. This not only pointed out the lack of regular supportive supervision that the facilities received from the CHMT, but also the lack of information on the supportive supervision policy details among the health workers that should be supervised. Other researchers have also identified also identified irregular supervisory visits as a problem with health systems in Africa [4, 7, 16, 17]. The SCHMT also conducted supervisory visits irregularly despite the apparent agreement by the respondents that they were expected to visit every quarter. This lack of regular supervisory visits was found to be comparable with the findings of other similar studies among health facilities in Africa [4, 16]. The lack of supervision consistency by the immediate level of supervision, the FHMT, was also demonstrated in the study, just as it had been similarly brought out in Ugandan and Zambian studies that looked at the levels of supportive supervision within health facilities that health workers received [18–20].

This study unearthed the lack of a regular supportive supervision visits that health facilities received in Kiambu County. These findings agreed with those of a similar study conducted in Zambia that examined the causes of poor performance of community health workers in the Kalabo district [20] and another study conducted in Tanzania on improving motivation among primary health care workers [7]. All levels of supervision were found not to be consistent with their visits, a scenario that contributed to the weak medicines management systems in government health facilities. However, some level of supportive supervision was periodically conducted among the health facilities as was demonstrated by 78.3% of the respondents who reported that they had had their most recent supervisory visit within the last month as of the study date implying that the study could have been carried out after a round of supportive supervision had just been done. The frequency of supervisory visits was found not be dependent on the travel distance to the health facilities for both the CHMT and the SCHMT. These findings were in contrast with the findings of other studies that identified the distance between the supervisors’ offices and the health facilities as a challenge, with facilities farther away receiving fewer visits and lower quality of supervision [1, 6, 8, 21]. Checklists for guiding supportive supervision existed but were not always used during the visits as has been observed in other similar studies [4, 20] and their use during supervision was independent of the tier of care of the health facility visited. This was in contrary to the findings of other studies that showed varying levels of the quality of supervision and the use of checklists at different tiers of care, with higher level health facilities receiving better quality supervision [22, 23].

Action Plans developed at the end of supervisory visits was poorly followed and this was partly responsible for the lack of implementation of tasks and decisions made during the previous visits. Other studies have also demonstrated this lack of follow up to action plans developed after supervisory visits and have attributed it to the perpetual weaknesses seen in African health systems, especially in rural settings [1, 24, 25]. The higher the tier of care of the health facility visited, the higher the likelihood that an Action Plan would be developed at the end of the supervisory visit. 44% of the chance to develop an Action Plan was explained by the level of care of the facility as demonstrated in other studies [22, 23]. Most respondents were not satisfied with the level of supervision for medicines management that they received despite how necessary they felt it was for improving medicines availability in the health facilities. These findings added to the high level of discontent that have been shown by the health care workers on the perceived level of supportive supervision that they receive from their supervisory teams [26–28]. Satisfaction with the level of supervision varied across the different cadres. More Pharmacists, than Pharmaceutical Technologists, Clinical Officers and Nurses felt that the medicines management supervisory visits that health facilities received were inadequate, while more Nurses felt that the visits were adequate. These findings showed the different levels of satisfaction at the workplace for the different health professionals as had been shown in studies in Uganda [29] and Pakistan [30]. Satisfaction also varied with the level of education of the respondents. Lower levels of education were associated with more contentment with the adequacy of the supervisory visits. This agreed with the findings of other studies which found out that more educated workers were relatively less satisfied and were more likely to rate systems badly [31, 32]. Contentment also varied with the tier of care from which respondents worked. Respondents from lower tier health facilities were associated with more contentment with the level of supportive supervision that they received. Higher tiers of care in Kenya have proportionally more specialists and higher educated health professionals [33] who rated the system harshly compared to lower tiers of care and thus the observed skew based on the tier of care of health facilities from which the respondents came from.

This study unearthed gaps in the supportive supervision for medicines management in Kiambu County, Kenya. It showed the lack of familiarity of the supportive supervision policy by the health workers, the failure by the supervisory teams to consistently use the tools for supervision, the inconsistency of the visits, and the level of discontent on the perceived level of supportive supervision by health workers in the county. The potential limitation of this study is that the study was conducted at a time when the country was in the process of devolving health services from the national government to county governments against the wish of most health workers in government health facilities and this could have had a confounding effect on the satisfaction ratings by the health workers.

## Conclusion

Supportive supervision by the health managers was not regularly conducted and most health workers did not know how often to expect supervisory visits from the different levels of management. The quality of the visits was also low since standard checklists were not always used despite their availability. There was lack of continuity in the supervisions since action plans were rarely followed up in the subsequent visits by the supervisory teams. Health workers managing medicines in the county were not satisfied with the level of supervision that they received from the different levels of health management in the county. A follow up study needs to be conducted from the health managers’ perspective in order to understand the actual gaps between policy and practice, and establish a sustainable way in which the supportive supervision for medicines management can be strengthened at the county level.
